# Access factors linked to maternal deaths in Lundazi district, Eastern Province of Zambia: a case control study analysing maternal death reviews

**DOI:** 10.1186/s12884-018-1717-1

**Published:** 2018-04-16

**Authors:** Nkumbula Moyo, Mpundu Makasa, Mumbi Chola, Patrick Musonda

**Affiliations:** 1 Macha Research Trust, Choma, Zambia; 2 Department of Public Health, School of Medicine, University of Zambia, Lusaka, Zambia

**Keywords:** Maternal deaths, Maternal death review, Access factors, Verbal autopsy, Delays

## Abstract

**Background:**

Access factors associated with maternal death are important to understand because they are considered to be an essential measure of women’s health and indicative of the performance of health care systems in any community globally. This study aimed to analyse the access risk factors linked to maternal deaths in Lundazi district of the Eastern Province of Zambia using secondary data obtained from maternal death reviews and delivery registers.

**Methods:**

This was a case-control study with cases being recorded maternal deaths for Lundazi district (*n* = 100) while controls were randomly selected Lundazi District Hospital deliveries (*n* = 300) for the period 2010 to 2015. STATA™ (Stata Corporation, Texas, TX, USA) version 12.0 was used to analyse data. Odds ratio and 95% confidence intervals with associated *p*-values were used to analyse disparities between cases and controls while bivariate and multivariate regression analyses were done to show associations.

**Results:**

The likelihood of experiencing maternal death was 94% less among women who completed their scheduled antenatal care visits than those who did not (OR 0.06, 95% CI = 0.01–0.27, *p* = < 0.001). Delayed referral associated with maternal deaths and complications were 30% (30) for cases, 12% (37) for controls and 17% (67) for both cases and controls. Long distances, unskilled deliveries were 3%, (15) for both cases and controls with 13% (13) for cases and 1% (2) for controls only.

**Conclusion:**

Antenatal care is important in screening for pre-existing risk conditions as well as complications in early stages of pregnancy that could impact adversely during pregnancy and childbirth. Delay in seeking health care during pregnancy could be minimised if health services are brought closer to the communities to reduce on distances covered by pregnant women in Lundazi. Maternal education appears to influence antenatal health care utilisation because greater knowledge and understanding of the importance of antenatal care might increase the ability to select most appropriate service. Therefore, there is need for Lundazi District Health Office to scale up interventions that motivate women to make at least four scheduled antenatal care visits during pregnancy as recommended by the World Health Organization.

**Electronic supplementary material:**

The online version of this article (10.1186/s12884-018-1717-1) contains supplementary material, which is available to authorized users.

## Background

Maternal death is death of a woman while pregnant or within 42 days of termination of pregnancy, irrespective of duration and site of pregnancy, from any cause related to or aggravated by the pregnancy or its management but not from accidental or incidental causes [1]. Under the Sustainable Development Goal (SDG) number 3.1, the target is that by 2030 the global maternal mortality ratio should have reduced to less than 70 per 100,000 live births for every country including Zambia [2]. While some districts may have done considerably well in reducing maternal mortality, Lundazi district in the Eastern province of Zambia is one of the districts still struggling to reduce maternal mortality to acceptable levels [3]. Results from the 2013–2014 Zambia Demographic and Health Survey (ZDHS) conducted by Central Statistical Office (CSO) in conjunction with Ministry of Health of Zambia indicate a national maternal mortality ratio of 398 per 100,000 live births. In other words, for every 1000 live births in Zambia, four women (3.98) died during pregnancy, during childbirth, or within 2 months of childbirth during the 7 years preceding the 2013–14 ZDHS [4].

Lundazi is one of the districts with high mortality rates because together with Nyimba district of Eastern Province of Zambia, maternal mortality ratio of 410 maternal deaths per 100,000 live births was estimated [3]. This is more than the national level maternal mortality ratio of 398 per 100,000 reported in the 2013/2014 ZDHS. However, information regarding local factors associated with maternal deaths is not readily obtainable. There has been no study conducted in the district to establish risk factors which are associated with maternal death yet such information is valuable in implementing and evaluating maternal health interventions.

Maternal mortality is an important measure of women’s health and indicative of the performance of health care systems [5]. Establishing local factors associated with maternal death for any community is a significant step in strengthening interventions to reduce maternal deaths [6]. It has also been observed that factors associated with maternal death are a sensitive indicator of the status of women, access to care, adequacy and quality of healthcare in developing countries [5].

The majority of the people of Eastern Province are poor with an overall incidence of poverty at 80% and extreme poverty at 66% [7]. The literacy levels of the people for Eastern Province remain quite low at 47% for women and 73% for men [4]. The population for the province mostly live in rural areas where the road network is poor and access to health facilities is a challenge [7]. According to Provincial Health Profile for 2011–2012, Lundazi district recorded the least antenatal coverage at 67% compared to 96% highest coverage recorded by another district of Chama district within Eastern Province of Zambia.

The 2012 Zambia National Health Policy estimates that 80% of Zambian population use traditional and alternative services for their day-to-day health care. In Eastern province of Zambia, it was established that cultural and traditional norms still persist in communities among women throughout the period prior to pregnancy, during pregnancy, nutrition, after delivery and during breastfeeding that affect uptake of antenatal care services [8]. These practices may put the health of pregnant women and their child at risk. Antenatal care is important for the maintenance of good health of a pregnant woman and the unborn child. WHO recommends four or more scheduled antenatal care visits as it improves maternal and neonatal outcome and reduce death. In resource limited communities, basic antenatal care is an important determinant of maternal mortality when practiced as one of the basic components of maternal health care [9].

Access to maternal health services, particularly facility childbirth remains challenging mainly in rural Zambia and this leads to delays in seeking care. A number of factors are attributed to challenges faced by public health officials in Zambia to effectively scale up outreach maternal health services and targeted messages to create awareness about maternal deaths. Zambia’s maternal health services have been weakened by high disease burden, low literacy levels, inadequate qualified health staff, poor transport network, lack of investment in maternity waiting shelters for expectant mothers to lodge in as they await child birth among other factors [10].

While scientific research on maternal mortality is focused mainly on clinical factors, this approach may not be most useful if we are to understand the problem of factors associated with maternal death [1]. This approach does not take in to account the importance of economic, political and social micro structural factors and other social determinants of maternal health [6]. It is important to note that while information generated from maternal death reviews and community verbal autopsies is available, it has not been prudently compiled and used to evaluate factors that are associated with maternal death in Lundazi. This study was conducted to determine access factors linked with maternal deaths in Lundazi utilizing secondary data collected through maternal death surveillance and response reports and routine birth and death registration information.

## Methods

The study was conducted in Lundazi at the District Health Office. It included information obtained from health facilities within Lundazi district that conduct deliveries and recorded maternal deaths during the period 2010 to 2015. Lundazi district is situated about 755 km by road to the eastern side of the Zambian capital city of Lusaka and has a population of 366,432 as projected from 2010 national census [4]. Maternal Death Surveillance and Response (MDSR) reviews are conducted every quarter of the year to examine factors and causes associated with maternal death. This process provides a platform for documenting what obstetricians, gynaecologists, midwives and doctors propose as factors and cause for maternal death [11]. It is from these review reports that maternal death information were extracted for analysis.

A case control study design was used where cases were all maternal deaths recorded by Lundazi District Health Office for the period 2010 to 2015. These were extracted from secondary data of MDSR reports and community verbal autopsies. Verbal autopsies are data collection tools used to assign cause of death through interviews with family or community members, where medical certification of cause of death is not available [12]. Data from verbal autopsies assists reviewers in summarising factors associated with a community maternal death and it is from this source that secondary data was extracted. The researchers were not involved in collection of data using the verbal autopsy. However, verbal autopsies were part of the source for the secondary data which was mined.

Controls were 300 (n) records of women who survived during the same period and randomly selected from the district hospital delivery register. This sample was obtained from a population of 2794 (N) complicated Lundazi district hospital deliveries conducted in the period 2010–2015 and met the selection criteria. We used a computer to randomly select every 9th record from the delivery register on an Excel spread sheet. Lundazi District Hospital is the main referral health facility for complicated obstetric cases but also ordinary deliveries from the surrounding communities. The ratio of cases to control sampled was 1:3.

All recorded maternal deaths in Lundazi district were considered for this study because maternal death is an event which is relatively rare. Lundazi District Hospital is a centrally located referral hospital where women from all corners of the district with obstetric complications deliver. Randomly selected controls had similar characteristics as cases and were drawn from the same population. In Zambia, it is estimated that 30–50% of all child births occur at home and most of the home deliveries are known to take place in rural than urban areas [7].

OpenEpi was used to estimate the sample size for the study with the following assumptions: unmatched case-control study with an estimated exposure rate among the controls of 30%. Comprehensive analysis of Demographic Health Surveys data from the 2000–2010 suggest that about 30% of pregnant women in the Sub-Saharan region do not complete their antenatal care attendance [13]. These specifications were expected to give a power of 80% and to detect odds ratio of 2.0 or greater, at a confidence level of 95%, with the alpha (α) level of 0.05, and case to control ratio of 1:3. The national risk rate is 398 deaths per 100,000 live births.

### Data analysis

Data extraction checklist was developed to extract relevant information from MDSR reports and verbal autopsies as well as hospital delivery registers. The data extracted was used to identify key characteristics of women who died of maternal cause during the period 2010 to 2015. Data was entered in Excel spread sheet, cleaned before exporting it to STATA version 12.0 (Stata™ Corporation, Texas, TX, USA) for analysis. Thirteen records in the register out of 2807 were not included in the sampling frame because the primary outcome of interest (died or survived) was not known or recorded.

**Fig. 1:**
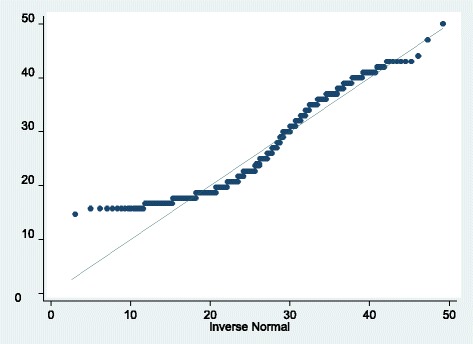
QQ plot testing for normality of distribution for participants’ age

Preliminary data analysis involved description of predictor variables to understand their distribution in relation to dependent variable (maternal death being the binary outcome). Antenatal care attendance as an exposure factor for both cases and controls as shown in Table 1 was summarised. Descriptive statistics, inter-quartile ranges, means, medians and standard deviations were analysed for continuous variables such as age, gestation and parity. Test for normality for age distribution of records of women analysed were done using QQ plot and normal distribution curve to determine if age distribution of the case and control populations had a common distribution pattern (Fig. 1). Frequency and percentage distributions for discrete variables were computed with cross tabulations to compare cases and controls. Logistic regression was used to analyse the dataset to describe the relationship between the dependent variable ‘maternal deaths’ with the selected predictor variables.

**Table 1:** Demographic characteristics with ANC attendance as exposure factor

Characteristic	Cases (*n* = 100)	Controls (*n* = 300)	Completed ANC (*n* = 400)
1. Maternal age			
Below 17 years of age	13 (13%)	52 (17%)	9 (14%)
17–34 years	50 (50%)	193 (64%)	140 (58%)
Above 34 years	37 (37%)	55 (18%)	35 (38%)
2. Marital status			
Married	88 (88%)	255 (85%)	174 (51%)
Not married	12 (12%)	45 (15%)	10 (18%)
3. Education level			
No formal education	34 (34%)	65 (22%)	17 (17%)
Primary education	55 (55%)	157 (52%)	98 (46%)
Secondary & above	11 (11%)	78 (26%)	69 (78%)
4. Educational level of participant’s husband			
No formal education	12 (12%)	18 (6%)	2 (7%)
Primary education	62 (62%)	115 (38%)	67 (38%)
Secondary & above	16 (16%)	122 (41%)	105 (76%)
No husband	10 (10%)	45 (15%)	10 (30%)
5. Religion			
Christian	90 (90%)	289 (96%)	172 (45%)
Muslim	8 (8%)	11 (4%)	12 (63%)
Others	1 (1%)	0 (0%)	2 (50%)
Year of record			
2010	18 (18%)	38 (13%)	24 (43%)
2011	19 (19%)	58 (19%)	38 (49%)
2012	21 (21%)	58 (19%)	36 (38%)
2013	18 (18%)	58 (19%)	29 (38%)
2014	15 (15%)	53 (18%)	30 (44%)
2015^a^	9 (9%)	35 (12%)	27 (61%)
6. Distance from health facility			
Within 5 Km radius	37 (37%)	135 (45%)	98 (57%)
Beyond 5 Km radius	63 (63%)	165 (55%)	86 (37%)

^a^Data collection for the year 2015 was only for 9 months compared to full calendar years for the other 5 years

## Results

A total of 400 records were reviewed (*n* = 100 for cases and *n* = 300 for controls) for the period January 2010 to September 2015. For cases, 18 (18%) deaths were recorded in 2010, 19 (19%) in 2011, 21 (21%) in 2012, 18 (18%) in 2013, 15 (15%) in 2014 and 9 (9%) in 2015. The mean age for all participants was 25.9 years with a standard deviation of 8.3. The youngest participant was 14 years while the oldest participant was 50 years. Twenty five per cent of participants were aged 19 years or less. Half of the participants (50th percentile) were equal to or below the age of 24 years. The age for the 3rd quartile (75%) were below or equal to 33 years. Table 1 shows demographic characteristics for cases and controls.

The likelihood of experiencing maternal death was 94% less among women who completed their scheduled antenatal attendance than those who never attended antenatal care (OR 0.06, 95% CI = 0.01–0.27, *p* = < 0.001). The frequency of women whose antenatal care attendance was complete up to their fourth scheduled visit for both cases and control groups was 184 (46%). Twenty five (6%) never attended ANC services during their pregnancy. However, among the cases, only 13 (13%) had complete scheduled ANC attendance. This is in comparison to 171 (57%) who completed their scheduled ANC attendance among the controls. Twenty two (22%) of cases and 3 (1%) of controls never attended ANC services during their pregnancy. Table 1 shows relationship between ANC attendance and other demographic characteristics for both cases and controls.

Delay in seeking maternal health increased the risk of maternal death (OR 4.4; 95% CI 2.24–9.15 and *p*-value < 0.0001). Two hundred and seventy six women out of 400 (69%) women delayed in seeking health services. Cases reported 88 (88%) of the women having delayed in seeking health services while among controls, 188 (63%) women delayed in seeking health care services. Delay in making decisions accounted for 163 (40%) of all delays with cases accounting for 52 (52%). Table 2 illustrates this relationship. Pre-existing disease conditions increased the risk of maternal death (OR 2.5; 95% CI = 1.40–4.30 and *p*-value = 0.0006). It was established that 163 out of 400 participants (41%) had pre-existing maternal risk conditions. Fifty one (51%) had pre-existing risk conditions among cases compared to 112 (37.4%) among the control group.

**Table 2:** Bivariate analyses showing factors associated with cases and controls

Characteristic	Cases (*n* = 100)	Controls (*n* = 300)	Total (*n* = 400)	OR (95% CI)	*p*-value
7. Presence or absence of risk condition				2.5 (1.40 - 4.30)	0.0006
Present	51 (51%)	112 (37%)	163 (41%)		
Absent	35 (35%)	186 (62%)	221 (55%)		
Not known	14 (14%)	2 (1%)	16 (4%)		
8. Antenatal Care (ANC) attendance				0.12 (0.06–0.22)	
Never attended ANC	3 (1%)	22 (22%)	25 (6%)		< 0.0001
1 or more ANC	126 (42%)	63 (63%)	189 (47%)		
Completed all visits	171 (57%)	13 (13%)	184 (46%)		
Attendance not known	0 (0%)	2 (2%)	2 (1%)		
9. Presence of complications				3.0 (1.73–5.36)	< 0.0001
Present	59 (59%)	93 (31%)	152 (38%)		
Absent	32 (32%)	207 (69%)	239 (60%)		
Not recorded or known	9 (9%)	0 (0%)	9 (2%)		
10. Any delay to seek health care				4.4 (2.24–9.15)	< 0.0001
Yes	88 (88%)	188 (63%)	276 (69%)		
No	12 (12%)	112 (37%)	124 (31%)		
11. Delay type					
Delay to make decision	52 (52%)	111 (37%)	163 (40%)		
Delay to reach facility	11 (11%)	66 (22%)	77 (19%)		
Delay to access service	25 (25%)	11 (4%)	36 (9%)		
12. Parity				2 (1.04–3.89)	0.0187
0–4 viable pregnancies	64 (64%)	241 (80%)	305 (76%)		
5–7 viable pregnancies	23 (23%)	43 (14.3%)	66 (17%)		
≥ 8 viable pregnancies	13 (13%)	16 (5.3%)	29 (7%)		
13. Gestational age				1.03 (0.69–1.52)	0.8973
9 months	66 (66%)	288 (96%)	354 (89%)		
Below 9 months	33 (33%)	11 (3.6%)	44 (11%)		
Above 9 months	0 (0%)	1 (0.25%)	1 (0%)		
14. Care time				1.02 (0.87–1.18)	0.8270
Within 2 h	30 (36%)	22 (11.7%)	52 (19)		
Within 24 h	30 (37%)	135 (77%)	175 (65%)		
After 24 h	22 (27%)	21 (11%	43 (16%)		
15. Sought traditional health care				3.6 (2.21–6.02)	< 0.0001
Yes	55 (55%)	86 (29%)	141 (35%)		
No	39 (39%)	210 (70%)	249 (62%)		
Not known	6 (6%)	4 (1%)	10 (3%)		
16. Marital status				1.3 (0.65–2.81)	0.2863
Married	88 (88%)	255 (85%)	343 (85%)		
Not married	12 (12%)	45 (15%)	57 (14)		
17. Education level of participants				0.5 (0.32–0.92)	0.0107
No formal education	34 (34%)	65 (22%)	99 (25%)		
Primary education	55 (55%)	157 (52%)	212 (53%)		
Secondary & above	11 (11%)	78 (26%)	89 (22%)		

Formal education reduced the risk of maternal deaths (OR 0.5; 95% CI = 0.32–0.92; *p* = 0.0107). Thirty four women (34%) had no formal education among cases compared to 65 (21.7%) among the controls. Only eleven (11%) women among the cases had formal education up to secondary level compared to 78 (26%) for the control group. Table 2 shows this relationship. Sixty nine (78%) of women who had secondary education or above also completed their antenatal care attendance. This is in comparison to 17 (17%) women with no formal education who did not complete their antenatal care attendance. This relationship was however not statistically significant (OR 2.1; 95% CI = 0.8–5.3; *p* = 0.0610) as shown in Table 1.

The relationship between antenatal care attendance and distance to the nearest health facility was statistically significant (OR = 2; 95% CI = 1.4–3.2; *p* = 0.003). Most cases, 63 (63%) lived beyond 5 Km from the nearest health facility compared to 165 (55%) for controls as shown in Table 1. Parity of greater than 8 increased the risk of maternal death by an odds ratio of 2 (95% CI = 1.04–3.89 and *p* = 0.0187). Thirteen women (13%) among cases had parity of greater than 8 while 16 (5%) of women among controls had parity of more than 8.

### Indirect causes for maternal deaths

Relative to controls, cases had 4.7 times the odds of having been affected by indirect causes for maternal deaths during pregnancy (95% CI = 2.83–7.95, *p* = < 0.0001). Table 3 outlines indirect causes of maternal deaths and how they affected both the case and control groups. Further analysis showed that women who presented with indirect causes for maternal deaths were 3 times more likely to die than those who did not have complications (95% CI = 1.73 to 5.38 and *p*-value = < 0.0001). Out of the 400 women assessed for presence of indirect causes of maternal deaths during pregnancy, 239 (60%) were established to have not been affected while 152 (38%) were affected. Among cases, 59 women (59%) were recorded to have been affected by indirect causes of maternal deaths while in the control group only 93 (31%) women were affected during their pregnancy.

**Table 3:** Indirect causes of maternal deaths affecting cases and controls

Indirect cause of deaths	Cases (*n* = 100)	Controls (*n* = 300)	Total
Haemorrhage	31 (31%)	49 (16%)	80 (20%)
Anaemia	14 (14%)	12 (4%)	26 (7%)
Infections	17 (17%)	12 (4%)	29 (7%)
Obstructed labour	9 (9%)	49 (16%)	58 (15%)
Eclampsia	7 (7%)	9 (3%)	16 (4%)
Abortion	7 (7%)	3 (1%)	10 (3%)
Others	12 (12%)	5 (2%)	17 (4%)
No complication	0 (0%)	101 (56)	101 (40)
Unknown cause	3 (3%)	0 (0%)	3 (1%)

It was established that 31 women (31%) among the cases died of indirect causes of maternal deaths arising from haemorrhage (postpartum or intra-partum). Seventeen women (17%) died as a result of other indirect causes arising from infections while 14 (14%) died of effects of anaemia. Obstructed labour accounted for 9 (9%) of indirect causes of maternal deaths among the cases and 49 (16%) among controls. Eclampsia and abortions accounted for 7% each of the recorded deaths. Other indirect causes accounted for 12% of maternal deaths recorded while indirect causes for 3% of maternal deaths were not recorded or were not known.

## Discussion

In this analysis of maternal death reviews and delivery records for Lundazi district as a case–control study, it was found that delay in seeking care by pregnant women was the leading factor associated with maternal deaths and complications. This finding is consistent with that of Shah et al. [6] who showed that delayed referrals to health facilities is associated with increased maternal death. He further summarised the delays associated with maternal deaths into three categories of ‘delays’ as: delay in the decision to seek care, delay in getting to a health facility and delay in the provision of adequate care. In this study, it was established that delay in making decisions was the most frequent type of delay followed by delay to reach facility and delay to access services respectively for both cases and controls albeit more among cases. The outcome that cases had more than four times the odds of having delayed to access maternal health services than the controls is comparable to the findings by Cham et al. (2005) who established that women who died of maternal cause were more likely to have delayed to receive prompt and adequate obstetric care at the hospital level [14]. Considering that the majority of women in this study lived beyond 5 km radius from the nearest health facility, failure to access maternal health services due to long distance, non-availability of transport and impromptu referral of critical cases could have contributed to the delays. The 3 delays framework was originally used to analyse a sample of 12 maternal deaths that occurred in a longitudinal cohort of pregnant Haitian women [15].

Antenatal care (ANC) attendance was another important factor which was associated with maternal death and complications in this study because women who surpassed the median number of visits, amounting to 2 or more, experienced lesser maternal complications and death. Our study showed that making more antenatal care visits was protective and beneficial. Adequate antenatal care services may also lead to women having improved uptake of skilled health facility delivery and attendance. For instance, in a nationally representative survey in Kenya by Mangeni et al. [16], it was shown that the number of ANC visits were significantly associated with skilled health facility delivery. However, in our study, it was not possible to determine the exact linear relationship between the number of antenatal care visits and the risk of maternal death because of the relatively small size of the study. The point that antenatal care was protective and beneficial did not imply we have evidence of relationship between antenatal care services and maternal death or complications in Lundazi.

Another important finding was that presence of a pre-existing high risk condition or illness such as anaemia, multiparity and previous caesarean section was significantly associated with increased maternal complications and maternal death. This finding is consistent with other studies in developing countries such as that by Yego et al. [17] who concluded that pre-existing high risk conditions are related to the effect of indirect obstetric causes on maternal death. A community-based study on the magnitude and causes of maternal mortality by Aggarwal et al. [18] showed that indirect causes of maternal death accounted for 27% of all maternal deaths and the most common indirect obstetric causes were anaemia. In our study, anaemia was ranked as the third indirect cause of maternal death at 14% (14) as established from the maternal death review reports for Lundazi district. This could be due to inadequate antenatal care attendance, an intervention which could have been used to prevent such conditions and ultimately maternal deaths.

Women with secondary or higher education were less likely to develop maternal complications or death. This is in agreement with Ngoc et al. [19] who reported a higher risk of maternal mortality among illiterate women. Similarly, Liang et al., [20] established that education is the only individual-level variable that is consistently a significant predictor of maternal health service utilization and consequently closely associated with maternal death. They also established that the odds of reporting use of antenatal care services increase steadily with education such that women with post-secondary education are five times as likely to report service use compared to their counterparts with no formal education. This finding is important because it stresses the role of education for women in acquiring and considering the benefits of good health. The benefits of education in maternal health includes antenatal attendance, family planning and being able to make appropriate decisions before, during and after pregnancy [21].

Parity was observed to be an important factor for maternal deaths and complications because cases had significantly greater number of women with high parity than controls. This finding is consistent with that for Simonsen et al. [22] who concluded that grand multiparity was dangerous because, in his study, maternal mortality increased steadily from the 5th to the 10th pregnancy. It is widely observed that grand multiparous women from rural areas tend not to complete their antenatal care visits hence having less access to maternal health services than women of lower parity. [10].

### Study limitations

While it was not possible to randomly select the cases (total sampling method or census was used), controls were randomly selected from the hospital delivery register. As was anticipated of secondary data, it was collected for purposes other than the objectives of this study. Therefore, certain variables such as socio-economic status of participants were not analysed because this aspect was missing from maternal death review reports and hospital delivery registers. Additionally, unreported or missed community maternal deaths may have compromised the validity of our study findings. However, strong community health structures in Lundazi such as the Safe Motherhood Action Groups (SMAGs) are known to be proactive in reporting maternal deaths to minimise the likelihood of missing information on maternal deaths. Results from this research may not be generalised because the study setting may not be comparable with the province. Nevertheless, the findings are a reflection of maternal death reviews and hospital delivery reports for Lundazi district for the period 2010 to 2015.

## Conclusion

Inadequate antenatal care, delay in accessing obstetric care and low literacy levels were established to be important factors contributing to maternal deaths in Lundazi district. Delay in accessing maternal health services for both cases and controls appear to be justified by long distances women travel to reach their nearest health facilities as it has been shown that most women live beyond the recommended 5 km radius from health facilities. Our findings on low literacy levels among cases supports the view that better maternal education might affect antenatal health care utilisation because greater knowledge and understanding of the importance of antenatal care might increase the ability to select most appropriate service. Furthermore, social and cultural beliefs and practices which some women in Lundazi strongly hold could influence their decision on antenatal health care utilisation. Maternal deaths could be minimised if all women at risk are reached with targeted messages to create awareness of the importance of antenatal care and the dangers of delaying to seek maternal health services. Improved understanding of the causes of maternal deaths through enhanced maternal death surveillance and response (MDSR), confidential enquires, and other methods of accounting for every maternal death will provide more information to plan targeted interventions. Therefore, there is need for Lundazi District Health Office to scale up interventions that motivate and allow women to make at least four scheduled visits during pregnancy as recommended by the World Health Organization. Scaling up outreach maternal health services and providing Health Posts closer to the community can greatly improve access to maternal health services and subsequently lead to a reduction in avoidable maternal deaths.

## Additional file

Additional file 1: The dataset used for analysis. (DTA 23 kb)

## Abbreviations

ANC: Antenatal Care
APH: Anti-partum Haemorrhage
CI: Confidence Interval
CSO: Central Statistics Office
DMO: District Medical Office
MDG: Millennium Development Goals
MDSR: Maternal Death Surveillance Review
MMR: Maternal Mortality Rate
PPH: Post-Partum Haemorrhage
RHC: Rural Health Centre
SDG: Sustainable Development Goals
UNICEF: United Nations International Children Emergency Fund
VA: Verbal Autopsy
WHO: World Health Organisation
ZDHS: Zambia Demographic and Health Survey
ZRHC: Zonal Rural Health Centre

## References

[1] Karlsen S, Say L, Souza JP, Hogue CJ, Calles DL, Gülmezoglu AM, Raine R (2011). The relationship between maternal education and mortality among women giving birth in health care institutions: analysis of the cross sectional WHO global survey on maternal and perinatal health. BMC Public Health.

[2] Hawkes S, Buse K (2016). Searching for the right to health in the sustainable development agenda: comment on rights language in the sustainable development agenda: has right to health discourse and norms shaped health goals?. Int J Health Policy Manag.

[3] Capps, J., McSmith, D., Chikotola, B., Dzekedzeke, K., and Chishimba, P. (2013). ‘Mid-Term Performance Evaluation of the USAID/Zambia,’ Integrated Systems Strengthening Program (ZISSP).

[4] Central Statistical Office (CSO) [Zambia], Ministry of Health (MOH) [Zambia], and ICF International (2014). ‘Zambia demographic and health survey 2013–14.’ Rockville, Maryland, USA: Central Statistical Office, Ministry of Health, and ICF International.

[5] Roberts CL, Cameron CA, Bell JC, Algert CS, Morris JM (2008). Measuring maternal morbidity in routinely collected health data: development and validation of a maternal morbidity outcome indicator. Med Care.

[6] Shah N, Hossain N, Shoaib R, Hussain A, Gillani R, Khan NH (2009). Socio-demographic characteristics and the three delays of maternal mortality. J Coll Physicians Surg Pak.

[7] Maimbolwa MC, Yamba B, Diwan V, Ransjö-Arvidson AB (2003). Cultural childbirth practices and beliefs in Zambia. J Adv Nurs.

[8] Kruk ME, Rabkin M, Grépin KA, Austin-Evelyn K, Greeson D, Masvawure TB, Sacks ER, Vail D, Galea S (2014). Big push’to reduce maternal mortality in Uganda and Zambia enhanced health systems but lacked a sustainability plan. Health Aff.

[9] Lukonga E, Michelo C. Factors associated with neonatal mortality in the general population: evidence from the 2007 Zambia demographic and health survey (ZDHS); a cross sectional study. Pan Afr Med J. 2015;20(1):2–5. 10.11604/pamj.2015.20.64.5616 PMC4450022 26090022

[10] Hazemba AN, Siziya S. Choice of place for childbirth: prevalence and correlates of utilization of health facilities in Chongwe district, Zambia. Med J Zambia. 2008;35(2):53–6.

[11] Evjen-Olsen B, Hinderaker SG, Lie RT, Bergsjø P, Gasheka P, Kvåle G (2008). Risk factors for maternal death in the highlands of rural northern Tanzania: a case-control study. BMC Public Health.

[12] Chisha Z, Larsen DA, Burns M, Miller JM, Chirwa J, Mbwili C, Winters AM (2015). Enhanced surveillance and data feedback loop associated with improved malaria data in Lusaka,’ Zambia. Malar J.

[13] Kinney MV, Kerber KJ, Black RE, Cohen B, Nkrumah F, et al. ‘Sub-Saharan Africa's mothers, newborns, and children: where and why do they die?’ PLoS Med. 2010;7: e1000294 [PMC free article] [PubMed]. 10.1371/journal.pmed.1000294 PMC2888581 20574524

[14] Cham M, Sundby J, Vangen S. ‘Maternal motarlity in rural Gambia: a qualitative study on access to emergency obstetric care,’ Reprod Health. 2005;2:3. (PMC free article) – PubMed. 10.1186/1742-4755-2-3 PMC1142340 15871743

[15] Barnes-Josiah D, Myntti C, Augustin A (1998). The “three delays” as a framework for examining maternal mortality in Haiti. Soc Sci Med.

[16] Mangeni JN, Mwangi A, Mbugua S, Mukthar V (2013). ‘Male involvement in maternal health care as a determinant of utilization of skilled birth attendants in Kenya;’ Calverton.

[17] Yego F, D’Este C, Byles J, Williams JS, Nyongesa P. ‘Risk factors for maternal mortality in a tertiary Hospital in Kenya: a case control study.’ BMC Pregnancy Childbirth. 2014;14:38 10.1186/1471-2393-14-38 PMID: 24447854. 10.1186/1471-2393-14-38 PMC3904405 24447854

[18] Aggarwal A, Pandley A, Bhattacharya B. ‘Risk Factors for maternal mortality in Delhi slums: a community based case control study.’ Indian J. Med Sci. 2007;61 No. 9, New Delhi. 17785888

[19] Ngoc N, Merialdi M, Abdel-Aleem H, Carroli G, Manorama P, Zavaleta N, Campodonico L, Ali M, Hofmeyr GJ, Mathai M (2006). Causes of stillbirths and early neonatal deaths: data from 7993 pregnancies in six developing countries. Bull World Health Organ.

[20] Liang J, Dai L, Zhu J, Li X, Zeng W, Wang H, Li Q, Li M, Zhou R, Wang Y (2011). Preventable maternal mortality: geographic/rural-urban differences and associated factors from the population-based maternal mortality surveillance system in China. BMC Public Health.

[21] Alam N (2000). Teenage motherhood and infant mortality in Bangladesh: maternal age: dependent effect of parity one. J Biosoc Sci.

[22] Simonsen SME, Lyon JL, Alder SC, Varner MW (2005). Effect of grand multiparity on intrapartum and newborn complications in young women. Obstet Gynecol.

